# Capecitabine in combination with either cisplatin or weekly paclitaxel as a first-line treatment for metastatic esophageal squamous cell carcinoma: a randomized phase II study

**DOI:** 10.1186/s12885-015-1716-9

**Published:** 2015-10-14

**Authors:** Su Jin Lee, Sungmin Kim, Moonjin Kim, Jeeyun Lee, Yeon Hee Park, Young-Hyuck Im, Se Hoon Park

**Affiliations:** Division of Hematology-Oncology, Department of Medicine, Samsung Medical Center, Sungkyunkwan University School of Medicine, Seoul, Korea

**Keywords:** Esophageal squamous cell carcinoma, First-line palliative chemotherapy, Capecitabine, Cisplatin, Paclitaxel

## Abstract

**Background:**

The aim of this study was to assess the efficacy and safety of a combination regimen of capecitabine plus cisplatin (CC) or capecitabine plus paclitaxel (CP) as a first-line treatment in patients with metastatic esophageal squamous cell carcinoma.

**Methods:**

Patients with recurrent or metastatic esophageal squamous cell carcinoma were enrolled in this open-label, phase II, randomized trial. Patients were assigned to either the CC arm (days [D]1–14 capecitabine 1000 mg/m^2^ twice daily + D1 cisplatin 75 mg/m^2^, every 3 weeks) or the CP arm (D1–14 capecitabine 1000 mg/m^2^ twice daily + D1, 8 paclitaxel 80 mg/m^2^, every 3 weeks). The primary endpoint of the study was response rate and secondary endpoints were progression-free survival (PFS), overall survival (OS), toxicity and quality of life.

**Results:**

A total of 94 patients were entered into this study between October 2008 and October 2012, 46 patients in the CC arm and 48 in the CP arm. Patients in both arms received a median of six cycles of treatment (range, 1–14) and the response rates were 57 and 58 % in the cisplatin and paclitaxel arm, respectively. With a median follow-up of 23 months, the median PFS was 5.1 months (95 % CI 4.0–6.2 months) in the cisplatin arm and 6.7 months (95 % CI 4.9–8.5 months) in the paclitaxel arm, whereas the median OS was 10.5 months (95 % CI 9.2–11.9 months) in the cisplatin arm and 13.2 months (95 % CI 9.4–17.0 months) in the paclitaxel arm. Patients in the cisplatin arm were more likely to experience neutropenia and thrombocytopenia, whereas patients in the paclitaxel arm had a higher frequency of neuropathy and alopecia. Quality of life was similar between treatment arms.

**Conclusions:**

Both CC and CP regimens were effective and well tolerated as a first-line treatment in patients with metastatic esophageal squamous cell carcinoma.

## Background

Esophageal cancer is the eighth most common cancer worldwide [1]. It is estimated that 2199 new cases and 1352 deaths from esophageal cancer occur annually in Korea [2]. Although there is variation in the predominant histological type among regions, the majority of esophageal cancers represent squamous cell carcinoma arising from the upper-and middle-third of the esophagus. A large majority of patients present with advanced disease that is beyond the scope of cure, and even after surgical resection with curative intent more than 50 % of patients will develop recurrence and/or distant metastases [3].

Despite the lack of evidence for a survival benefit associated with cytotoxic chemotherapy, administration of fluoropyrimidine with or without cisplatin remains a common treatment strategy for patients with metastatic esophageal cancer. Similar to the results of clinical trials performed in patients with adenocarcinoma arising from the esophagus or stomach [4], a combination chemotherapy regimen of 5-fluorouracil and cisplatin was associated with response rates of up to 45 % in patients with squamous cell esophageal cancer [5, 6]. There have been efforts to substitute infusional 5-fluorouracil with oral fluoropyrimidines for improved tolerability and patient convenience. Our own phase II study reported the efficacy and tolerability of capecitabine plus cisplatin (CC) in chemotherapy-naïve patients with metastatic esophageal squamous cell carcinoma; the response rate was 58 % and the median overall survival (OS) was 11.2 months [7].

Among many chemotherapeutic agents, taxanes (paclitaxel or docetaxel) have been investigated as single agents or as combination therapy in this setting. When combined with cisplatin, paclitaxel is associated with a response rate of 40–50 % [8, 9]. Paclitaxel has also been suggested as a partner for capecitabine-based chemotherapy because taxanes upregulate the activity of thymidine phosphorylase (TP) [10], which is an essential enzyme for the activation of capecitabine. In addition to the possible synergistic effect of a capecitabine and paclitaxel (CP) combination, these agents have non-overlapping toxicities and therefore can be combined with reasonable tolerability. Combination chemotherapy with taxane plus capecitabine has been tested in a number of clinical trials and showed favorable efficacy and safety [11, 12]. Previous studies that explored different combination regimens involving capecitabine prompted us to design this randomized phase II trial in order to evaluate the efficacy and safety of CC and CP regimens in patients with metastatic esophageal squamous cell carcinoma who had not been previously treated.

## Patients and methods

This was an open-label, single-center, randomized, parallel phase II study (ClinicalTrials.gov NCT00816634). The protocol was reviewed and approved by the institutional review board at Samsung Medical Center ((#200807059, Seoul, Korea) and the trial was conducted in accordance with the Declaration of Helsinki. Patients with recurrent or metastatic squamous cell carcinoma of the esophagus who had not previously been treated palliative chemotherapy for metastatic disease were eligible. Patients were required to have at least one measurable metastatic lesion as defined by the Response Criteria in Solid Tumors (RECIST) v1.0, an Eastern Cooperative Oncology Group (ECOG) performance status of 0–2, a life expectancy of at least 3 months, and adequate hematologic (neutrophil count ≥ 1500/mm^3^, platelet count ≥ 100,000/mm^3^, hemoglobin ≥ 9.0 g/dl), renal (serum creatinine ≤ 1.5 mg/dl or creatinine clearance ≥ 50 ml/min) and liver function (bilirubin ≤ 1.5 mg/dl, AST/ALT ≤ 3 times the upper normal limit). Patients with no prior chemotherapy or only adjuvant chemotherapy that had been completed more than 6 months before registration, and no radiotherapy within 4 weeks before study registration were eligible. Patients who had other types of esophageal cancer tumors, central nervous system metastasis, severe comorbid illness, or active infection, and women who were pregnant or lactating, were excluded from the study. The nature of the study was fully discussed with the patients before the initiation of treatment, including an explanation of the risks and possible discomfort, as well as the potential benefits, and written informed consent was obtained.

Eligible patients were stratified by their ECOG performance status (0–1 vs. 2), and randomly assigned to receive CC (capecitabine 1000 mg/m^2^ orally twice a day on days 1–14 plus 75 mg/m^2^ of cisplatin intravenously on day 1) or CP (capecitabine as for CC plus 80 mg/m^2^ of paclitaxel intravenously on days 1 and 8). An identical dose regimen of capecitabine was used for both treatment arms. Study treatment was repeated every 3 weeks until documented disease progression, unacceptable toxicity, or patient refusal. Supportive care, including adequate pre- and post-hydration for patients in the CC arm and corticosteroids for patients in the CP arm, was provided according to guidelines. The use of hematopoietic growth factors was not allowed during treatment, except for patients with febrile neutropenia or grade 4 myelosuppression at the investigators’ discretion. Each cycle of chemotherapy was given if the patient’s blood counts had returned to normal and non-hematologic toxicities had resolved. All patients received chemotherapy as outpatients. After combination chemotherapy failed, second-line chemotherapy was recommended to all patients if their performance status was preserved.

The dosage of subsequent cycles was adjusted according to the toxic effects that developed during the preceding cycle. Baseline evaluation included a complete medical history and physical examination, blood counts, serum chemistry, chest x-ray, and chest computed tomography (CT) scan. Follow-up history, physical examination and toxicity assessment were performed before each 3-week cycle of treatment. Toxicity grading was based on the National Cancer Institute criteria (NCI-CTCAE version 3). The first evaluation with imaging was performed 6 weeks after the start of study treatment. Response was evaluated according to the RECIST criteria and was assessed by chest CT or by the same tests that were initially used to stage the tumor. In case of complete radiologic response, endoscopic evaluation of the primary tumor, if present, was mandatory. Progression in non-measurable lesions that led to deterioration of patient status was classified as progressive disease regardless of the status of the measurable lesions. We also assessed quality of life (QOL) using the EORTC-QLQ-OES18, which contains four scales that address dysphagia, eating difficulties, reflux, and esophageal pain, and six single items for problems with coughing, dry mouth, taste, choking when swallowing, speech, and swallowing saliva. These self-administered questionnaires were completed by patients at baseline, every two cycles, and at the end of treatment. QOL scores were descriptively recorded as baseline values and changes from baseline. As a general criterion for clinically significant improvement or deterioration, we defined a difference of ten or greater from baseline mean score as a clinically significant change.

The primary objective of this study was to assess the response rate in both treatment arms. Secondary objectives included assessment of PFS, OS, toxicity and QOL. This randomized phase II trial was statistically treated as two simultaneous phase II studies and the Simon’s two-stage optimal design was applied separately for each treatment arm [13]. A sample size of 94 patients was required to accept the hypothesis that the true response rate in each arm was greater than 40 % with 80 % power, and to reject the hypothesis that the response rate was less than 20 % with 5 % significance. In the first stage, if there were fewer than four responses out of the initial 13 patients for each group, early termination of the study was required. PFS and OS were estimated according to the Kaplan-Meier method, and the changes in QOL scores were calculated with a paired *t*-test. Since the study was designed to assess chemotherapy outcomes for two regimens simultaneously, exploratory analyses of efficacy were carried out using the Cox regression model. All data were analyzed using R for Windows software (version 2.11.1, http://www.r-project.org).

## Results

**Fig. 1:**
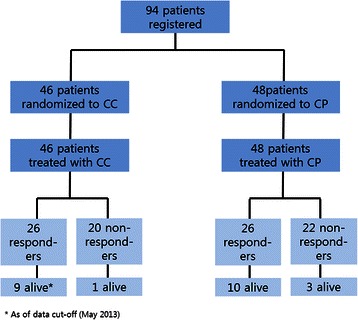
Flow diagram of all registered patients

Between October 2008 and October 2012, a total of 94 patients with esophageal squamous cell carcinoma were registered for the study. After randomization, 46 patients were allocated to the CC arm and 48 patients to the CP arm (Fig. 1). The median age was 63 years (range 34–82 years) and the majority of patients were male (98 %) and smokers (92 %). All patients had histologically proven squamous cell carcinoma of the esophagus. Fifty-nine patients had primary advanced disease and the remaining 35 had recurrent disease after prior surgery or definitive chemoradiation. The most common site of metastasis was supraclavicular or cervical lymph node (54 %), followed by abdominal lymph node (45 %), lung (32 %), bone (18 %), and liver (13 %). Baseline characteristics were available for all patients and are listed in Table 1.

**Table 1:** Patient characteristics

	Total (*n* = 94)	CC (*n* = 46)	CP (*n* = 48)
Age, years			
Median (range)	63 (34–82)	62 (46–76)	63 (34–82)
Gender			
Male	92	45	47
Female	2	1	1
Smoking history			
Never	7	5	2
Current or former	85	39	46
Prior therapy			
Surgery	31	15	16
Radiotherapy	20	10	10
Chemotherapy (adjuvant or neoadjuvant)	19	9	10
Initial disease status			
Initial advanced state	59	29	30
Recurred state	35	17	18
Locoregional disease	4	3	1
Distant metastasis	90	43	47
No. of metastatic sites			
One	45	24	21
Two or more	49	22	27
Metastatic sites			
Neck, supraclavicular LN	51	24	27
Abdominal LN	42	23	19
Lung	30	10	20
Bone	17	8	9
Liver	12	6	6

*CC* capecitabine plus cisplatin, *CP*, capecitabine plus paclitaxel, *ECOG*, Eastern Cooperative Oncology Group, *LN*, lymph nodes

### Treatment and adverse events

A total of 230 cycles of CC (median 6, range 1–12) and 278 cycles of CP (median 6, range 1–14) were delivered. Among the 94 patients who started study treatment, the main reasons for discontinuing treatment in the CC and CP arms included progressive disease (30 % versus 33 %), toxicity (9 % versus 13 %), and patient refusal (20 % versus 10 %). For patients treated with CC, the median dose intensities of capecitabine (573 mg/m^2^/week) and cisplatin (22 mg/m^2^/week) corresponded to 86 and 90 % of the scheduled doses. In the CP arm, the median dose intensities of capecitabine and paclitaxel were 596 mg/m^2^/week and 48 mg/m^2^/week, respectively (90 % of the scheduled doses). The median duration of therapy was 4.5 months and 5.8 months in the CC and CP arms, respectively (*P* = 0.064).

There was no significant difference in the occurrence of overall grade 3 or 4 adverse events between the two arms. The toxicity profiles are presented in Table 2. The most common grade 3 or 4 adverse event was neutropenia, which occurred in 16 patients (40 %) in the CC arm and 18 patients (38 %) in the CP arm. All grades of neutropenia and thrombocytopenia were more frequently observed in the CC arm, whereas peripheral neuropathy, myalgia, and alopecia were more common in the CP arm. Interstitial pneumonitis occurred in two patients in the CP arm. This event, which resolved after weeks of corticosteroid therapy, was possibly related to study chemotherapy. Three patients died of causes for which a relationship to the study treatment could not be completely ruled out. One death as a result of tumor bleeding occurred in the middle of the first CC cycle. Another patient died of neutropenic sepsis after receiving a fifth CP cycle. The third patient, who was known to have multiple lung and liver metastases, died of respiratory failure after 10 months of clinical response to CP. Since the tumor extent remained unchanged, the possibility of drug-related pneumonitis was not completely excluded.

**Table 2:** Safety of the study regimens

	CC		CP	
	Grade 1-2	Grade 3-4	Grade 1-2	Grade 3-4
Anemia	30	6	38	5
Neutropenia	16	16	9	18
Thrombocytopenia	24	2	7	1
Fatigue	14	0	11	1
Myalgia	0	0	23	1
Anorexia	26	0	29	2
Nausea	21	1	18	1
Vomiting	8	1	4	0
Stomatitis	11	0	20	2
Diarrhea	8	1	11	1
Constipation	6	0	5	0
Neuropathy	13	0	26	0
Alopecia	1	0	10	0
Skin	15	0	16	1
Nail changes	6	0	8	0

### Efficacy

Of the 94 patients, 11 could not be evaluated for clinical response because of early discontinuation of therapy. In an intent-to-treat principle, these patients were considered to have progressive disease.

The response rate was 57 and 58 % in the CC and CP arms, respectively. Additionally, 10 (22 %) CC and 12 (25 %) CP patients had stable disease (Table 3). Therefore, disease control (response plus disease stabilization) was achieved in 78 and 83 % of patients treated with CC and CP, respectively. Two CP patients exhibited complete response to chemotherapy and discontinued study treatment for resection with curative intent. The median duration of response in the 54 responders was 6 months (range 1–38 months): 4 months in the CC arm (range 1–31 months) and 7 months in the CP arm (range 1–38 months). Although not specified in the protocol, second-line chemotherapy was offered to 49 and 48 % of patients in the CC and CP arms, respectively, after failure.

**Table 3:** Treatment response

	Total (n = 94)	CC (n = 46)	CP (n = 48)
Best response			
Complete response	2 (2 %)	0 (0 %)	2 (4 %)
Partial response	52 (55 %)	26 (57 %)	26 (54 %)
Stable disease	22 (23 %)	10 (22 %)	12 (25 %)
Progressive disease	7 (7 %)	3 (7 %)	4 (8 %)
Not evaluable	11 (12 %)	7 (15 %)	4 (8 %)
Overall response rate	54 (57 %)	26 (57 %)	28 (58 %)
Disease control rate	76 (81 %)	36 (78 %)	40 (83 %)

**Fig. 2:**
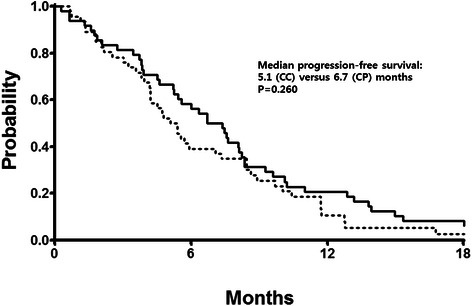
Progression-free survival for the capecitabine plus paclitaxel arm (CP, solid line) and capecitabine plus cisplatin arm (CC, dotted line)

**Fig. 3:**
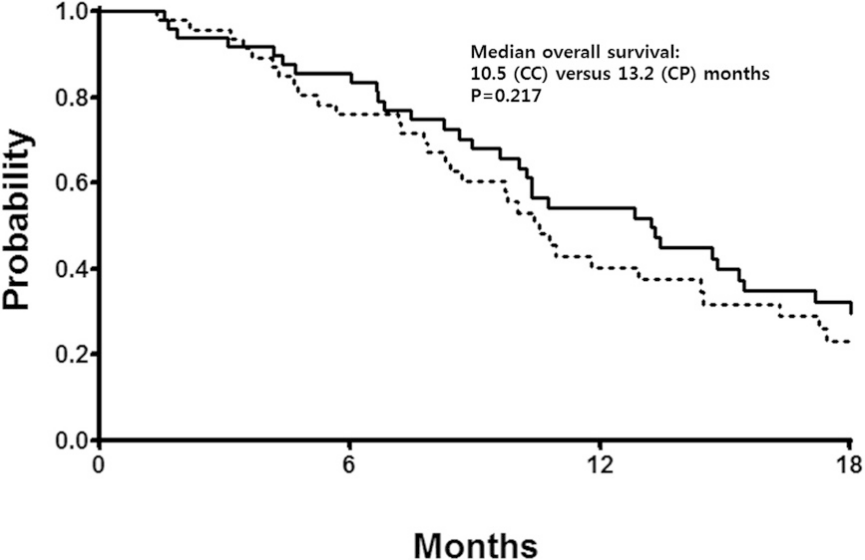
Overall survival for the capecitabine plus paclitaxel arm (CP, solid line) and capecitabine plus cisplatin arm (CC, dotted line)

With a median follow-up duration of 23 months, the median PFS was 5.1 months (95 % confidence interval [CI] 4.0–6.2 months) in the CC arm and 6.7 months (95 % CI 4.9–8.5 months) in the CP arm (Fig. 2). The median OS was 10.5 months (95 % CI 9.2–11.9 months) in the CC arm and 13.2 months (95 % CI 9.4–17.0 months) in the CP arm (Fig. 3). These differences were not statistically significant (log-rank *P* = 0.260 for PFS and *P* = 0.217 for OS). Analysis with the Cox proportional hazards model suggested that symptomatic disease at baseline (*P* = 0.012) and multiple sites of metastases (*P* = 0.028) affected the risk of progression or death (Table 4). In the multivariate model using these two parameters that showed individual prognostic value, significant interaction was noted between the number of metastatic sites and the ECOG performance status. The final model, incorporating the interaction terms, revealed that only ECOG performance status of 1 or higher remained significant (hazard ratio 2.075, 95 % CI 1.025–4.202, *P* = 0.043). There was no evidence of any significant heterogeneity in treatment effect according to the baseline characteristics (data not shown).

**Table 4:** Results of Cox model for progression-free and overall survival

		Progression-free survival			Overall survival		
		HR	95 % CI	*P*	HR	95 % CI	*P*
Age	≤60 years	1					
	>60 years	1.484	0.969-2.272	0.070	1.236	0.770-1.984	0.379
Smoking	Never						
	Ever	1.080	0.496-2.353	0.846	1.076	0.465-2.494	0.864
Performance status	ECOG 0						
	ECOG 1-2	1.110	0.661-1.862	0.694	2.381	1.215-4.673	0.012
Prior surgery	No						
	Yes	0.799	0.512-1.246	0.322	0.608	0.361-1.024	0.061
Prior radiotherapy	No						
	Yes	0.924	0.555-1.541	0.763	0.741	0.398-1.379	0.345
Prior chemotherapy	No						
	Yes	0.839	0.440-1.602	0.595	1.111	0.577-2.410	0.752
No. of metastases	One						
	≥Two	1.181	0.777-1.795	0.436	1.698	1.059-2.717	0.028
Lung metastasis	No						
	Yes	1.119	0.624-1.764	0.858	1.201	0.701-2.012	0.488
Liver metastasis	No						
	Yes	1.235	0.650-2.347	0.520	1.397	0.497-1.838	0.544
Treatment	CC						0.219
	CP	0.786	0.515-1.198	0.263	0.745	0.465-1.192	

*CC* capecitabine plus cisplatin, *CP* capecitabine plus paclitaxel

### Quality of life

Baseline QOL questionnaires were completed by 93 (99 %) patients. The scores for the baseline QOL were similar in both arms. The compliance of subsequent QOL questionnaires decreased to 40 % at the end of treatment. Over the whole study treatment period, there was no relevant difference between the study arms in the proportion of patients reporting QOL changes from baseline to post-treatment. Symptom scales also were similar between arms, except that reflux improved after CC chemotherapy and dry mouth was aggravated after CP treatment.

## Discussion and conclusions

In this phase II randomized study evaluating combination chemotherapy with capecitabine plus either cisplatin (CC) or weekly paclitaxel (CP) in patients with metastatic squamous cell carcinoma of the esophagus, the response rates were comparable in each arm (57 % for CC versus 58 % for CP), demonstrating that the two regimens were similar in terms of efficacy. This was also the case for median PFS and OS (5.1 months and 10.5 months, respectively, for the CC arm versus 6.7 months and 13.2 months, respectively, for the CP arm). Although the comparison between arms was performed for exploratory purposes, these observations suggest that platinum-free combination chemotherapy incorporating paclitaxel has an activity similar to that of platinum-based chemotherapy in terms of PFS and OS. Treatment was well tolerated with manageable toxicity profiles. The incidence and severity of toxicity was comparable to that previously reported [7, 14]. Although the compliance of patients in the QOL analysis was generally poor for both arms (40 % at the end of treatment), QOL showed similar results between the arms. The low compliance rate makes interpretation of the results difficult and raises the risk of bias as a result of patients with more severe illness being less able to complete QOL questionnaires or staff being less willing to approach them.

The best choice of chemotherapy regimen for patients with metastatic esophageal squamous cell carcinoma remains a matter of controversy and requires further investigation. The overall response rate of 57 % and median PFS of 5.1 months obtained with the CC arm are consistent with those reported in our previous phase II study that applied the same regimen [7]. The rationale for designing the present phase II trial to compare CC with CP was the potential for improved antitumor activity and tolerability. 5-FU has been considered a fundamental agent in the treatment of gastrointestinal cancers, and several studies have shown that 5-FU can be safely replaced by capecitabine without reducing the efficacy [15, 16]. A milestone phase III study of 316 patients with gastric cancer demonstrated that the capecitabine arm was not inferior to the 5-FU arm in terms of PFS (5.6 months versus 5.0 months) and OS (10.5 months versus 9.3 months) [17]. Based on studies performed with patients with gastric or esophageal adenocarcinoma, capecitabine is often used in combination with cisplatin [18]. However, it is well known that cisplatin is associated with significant toxicity and usually requires a high level of clinical monitoring and supportive care. There are several ways to circumvent cisplatin-related safety issues, including omitting the cisplatin or replacing it with a cytotoxic drug with similar activity. Among others, oxaliplatin has been actively investigated as a means to improve the efficacy and tolerability of capecitabine-based chemotherapy [19, 20]. The use of taxanes in combination with capecitabine merits attention. Lorenzen et al. [21] reported that patients achieved an overall response rate of 46 % with capecitabine plus docetaxel first-line chemotherapy. Yun et al. [14] also reported a high response rate of 75 % with a combination of capecitabine plus paclitaxel in this setting. Considering these findings, it was anticipated that a further improvement in efficacy and tolerability might be achieved by the substitution of cisplatin with weekly paclitaxel. In the present study, an identical dose regimen of capecitabine was used for both arms. Thus, the administration of cisplatin or paclitaxel was the only variable between the two treatment arms.

The results of the present phase II study should be interpreted with caution. We do not intend for these data to be interpreted as stating that one regimen is better than another. It should be kept in mind that only a small group of patients with metastatic squamous cell esophageal carcinoma was represented in this study, and the study was not adequately powered to compare the two treatment arms. Although the toxicity profiles were generally predictable and manageable, three deaths occurred that were possibly related to treatment. Of note, one patient died of respiratory failure while his metastatic lung disease remained stable. Pulmonary toxicity has been reported with taxane-based chemotherapy [22], and can sometimes be severe and even fatal. In the current study, interstitial pneumonitis developed in two patients in the CP arm although fortunately it was successfully treated with corticosteroid therapy. Considering the risk of such toxicities, our overall interpretation of these results is that the combination of CP does not warrant further studies in light of the non-hematologic toxicities. Given the comparable efficacy results, CC might be a reasonable first-line chemotherapy regimen for patients with metastatic esophageal squamous cell cancer. Other active and tolerable agents are becoming available and it is conceivable that the addition of molecularly-targeted agents to CC chemotherapy could improve the efficacy for treating esophageal squamous cell cancer without compromising tolerability.
